# Supplementary material for: Complexome profiling of the Chlamydomonas psb28 mutant reveals TEF5 as an early PSII assembly factor
Source: Plant Cell. 2025 Mar 17;37(6):koaf055. doi: 10.1093/plcell/koaf055 (PMC12164590; doi:10.1093/plcell/koaf055)
Supplement: koaf055_Supplementary_Data [file koaf055_supplementary_data.zip › psb28_interactive.zip!/plots/intensity/qualityPlots/peptides/_Cre10.g418450.t1.2.html]

### Cre10.g418450.t1.2

Trivial name:   
SeqCoverage [median]: 62.20  
Mass: 8.89 kDa  
  
 

| Sequence | MissedCleavage | Mass [Da] | wtIntensity | muIntensity | Score |
| --- | --- | --- | --- | --- | --- |
| HDPEQTAGITPETAEAAK | 0 | 1864.88 | 12567.77 | 21030.67 | 151.31 |
| KGDQFKNSIR | 2 | 1191.64 | 5831.90 | 8622.21 | 127.83 |
| SMFVGR | 0 | 695.34 | 7417.27 | 2434.59 | 115.83 |
| STSLFDNDMSK | 0 | 1243.54 | 56761.05 | 58764.23 | 195.40 |
| STSLFDNDMSKFVK | 1 | 1617.77 | 3546.63 | 5549.85 | 114.56 |
| VIRHDPEQTAGITPETAEAAK | 1 | 2233.13 | 30932.98 | 16813.22 | 77.96 |
|  |  |  | 117057.6 | 113214.8 |  |
